# Systemic Long-Distance Signaling and Communication Between Rootstock and Scion in Grafted Vegetables

**DOI:** 10.3389/fpls.2020.00460

**Published:** 2020-05-05

**Authors:** Xiaohong Lu, Wenqian Liu, Tao Wang, Jiali Zhang, Xiaojun Li, Wenna Zhang

**Affiliations:** Beijing Key Laboratory of Growth and Developmental Regulation for Protected Vegetable Crops, China Agricultural University, Beijing, China

**Keywords:** hormone transport, long-distance signaling, phloem transport, protein transport, RNA transport, systemic signaling, vegetable grafting, xylem transport

## Abstract

Grafting is widely used in fruit, vegetable, and flower propagation to improve biotic and abiotic stress resistance, yield, and quality. At present, the systemic changes caused by grafting, as well as the mechanisms and effects of long-distance signal transport between rootstock and scion have mainly been investigated in model plants (*Arabidopsis thaliana* and *Nicotiana benthamiana*). However, these aspects of grafting vary when different plant materials are grafted, so the study of model plants provides only a theoretical basis and reference for the related research of grafted vegetables. The dearth of knowledge about the transport of signaling molecules in grafted vegetables is inconsistent with the rapid development of large-scale vegetable production, highlighting the need to study the mechanisms regulating the rootstock-scion interaction and long-distance transport. The rapid development of molecular biotechnology and “omics” approaches will allow researchers to unravel the physiological and molecular mechanisms involved in the rootstock–scion interaction in vegetables. We summarize recent progress in the study of the physiological aspects (e.g., hormones and nutrients) of the response in grafted vegetables and focus in particular on long-distance molecular signaling (e.g., RNA and proteins). This review provides a theoretical basis for studies of the rootstock–scion interaction in grafted vegetables, as well as provide guidance for rootstock breeding and selection to meet specific demands for efficient vegetable production.

## Introduction

Grafting is one of the main means of asexual reproduction of horticultural crops such as fruit trees, vegetables, and flowers. It is used extensively to ameliorate the biotic stress, such as that caused by fungi and viruses, of crops (Cohen et al., 2000, 2005, 2007; Ioannou, 2001; Nisini et al., 2002), enhance abiotic stress tolerance to extreme temperatures and other environmental factors (Zhou et al., 2007). Grafting can also induce alterations in fruit traits, such as size (Garcia-Lozano et al., 2020), rind thickness (Fredes et al., 2017), and flesh firmness (Bertucci et al., 2018).

The mechanisms regulating the rootstock–scion interaction can affect plant growth and development, adaptation to environmental conditions, and physiological and biochemical characteristics. For example, grafting can result in an improvement of vegetable quality by increasing the synthesis of endogenous hormones and the acquisition and transport of mineral nutrients (Lee et al., 2010; Wang Q. et al., 2017); it can also alter secondary metabolites, creating novel flavors (Lee et al., 2010; Kakizaki et al., 2017), and the concentrations of fruit metabolites such as lycopene, carotenoids, and amino acids (Lee et al., 1996; Davis et al., 2008).

Recent advances in molecular biotechnology and bioinformatics techniques have stimulated research into the dual interaction between rootstocks and scions (Warschefsky et al., 2016). In vegetables, grafting and “omics” analysis have been integrated to examine the transport of mRNAs, small RNAs [specifically small interfering RNAs (siRNAs)], and proteins (Kim et al., 2001; Haywood et al., 2005; Zhang K. et al., 2014). In addition, grafting, especially in horticultural plant propagation, is an important method and a means to study basic processes such as flowering mechanisms, and long-distance conduction in plants (Turnbull, 2010; Goldschmidt, 2014). In these experiments, different grafting combinations of mutant and wild-type materials are subjected to some treatment, and the substance of interest (e.g., plant hormone, mRNA, protein) is detected in the rootstock and scion.

Here, we summarize research over the past two decades on grafting-induced changes in and transport of endogenous substances, including phytohormones, mineral elements, RNAs, and proteins, in vegetables. We pay particular attention to the potential molecular mechanisms underlying long-distance trafficking of RNAs and proteins between rootstocks and scions. We supplement our discussion with information on signaling transport verified in other plants and in non-grafting experiments. Our purpose is to provide a perspective from which to unravel the mechanisms underlying long-distance signaling between vegetable rootstocks and scions, and to provide a reference for the selection of vegetable grafting combinations.

## Physiological Aspects of the Rootstock–Scion Interaction in Grafted Vegetables

### Phytohormones

Current research on phytohormones is mainly focused on physiological function, biosynthesis, and metabolism, and signal perception and transduction (Li and Li, 2019). *Arabidopsis thaliana* (Arabidopsis), *Oryza sativa* L. (rice), and *Gossypium* (cotton) are most often used in this research. This work has laid a solid foundation for exploring phytohormone transport between the rootstock and scion in grafted vegetables.

Phytohormones can function either at their site of synthesis or can be transported through the vascular system to tissues relatively far from their source (Li and Li, 2019). Indole acetic acid (IAA; an auxin), cytokinins (CKs), and jasmonic acid (JA) have been shown to undergo polar transport between rootstocks and scions. For instance, red light sensed by leaves of normal tomato scions activates the synthesis of IAA (Guo et al., 2016), which is transported downward and promotes the normal development of lateral roots of diageotropica (*DGT*) mutant rootstocks whose organogenesis of lateral roots be abolished (Ivanchenko et al., 2015). In Arabidopsis, CKs and gibberellic acid (GA) can promote scion branch growth (Ko et al., 2014) and internode elongation (Regnault et al., 2015), respectively, through xylem transport (Matsumoto-Kitano et al., 2008; Kiba et al., 2013; Osugi et al., 2017) from root to shoot. Under osmotic stress, cotton roots induce leaves to synthesize a large amount of JA and transport it to the roots, which increases the content of PIP protein in the roots, enhancing their water absorption capacity (Luo et al., 2019). Furthermore, JA biosynthesis is induced by leaf injury, which causes shoot-to-root transport (Gasperini et al., 2015); conversely, carlactone (CL) can be transported upward from the rootstock to the scion, where it is converted into active strigolactone (SL) (Booker et al., 2005). Abscisic acid (ABA) in tomato rootstocks may be transported upward to regulate stomatal closure of scions (Dodd et al., 2009). Furthermore, GA may be produced tomato rootstocks and scions under drought stress (Gaion et al., 2018). However, further investigation using other methods, such as transgenic sequencing and RNA-seq, is needed to determine whether the phenotypic compensation, in mutants whose gene related to phytohormone transport was interfered, caused by grafting is a result of the transport of phytohormones between rootstocks and scions.

Interaction between phytohormones has been widely reported: (i) in biosynthesis – some genes encoding members of the auxin transcription factor ARF family can regulate CK biosynthesis by binding to the promoter of the adenylate isopentenyltransferase 5(*IPT5*) (Cheng et al., 2013) or the oxidase gene *OsCKX4* (Gao et al., 2014). In addition, several reports have confirmed that other phytohormones [JA, ABA, ethylene (Eth), GA] influence IAA biosynthesis (Cui et al., 2005; Cai et al., 2014; Zhao et al., 2014; Miao et al., 2018. (ii) In function—IAA and GA can regulate the negative gravity response of rice stem by antagonizing the expression of *XET* (Cui et al., 2005), brassinosteroids (BRs) interact with IAA and phosphatidylinositol signals to regulate vascular morphogenesis in Arabidopsis cotyledons (Lin et al., 2005), in tomato, the ratio of CK/ACC (positively) and ACC/ABA (negatively) is positively and negatively related to leaf growth and photosystem II (PSII) efficiency, respectively (Albacete et al., 2009). (iii) In signal transduction – in Arabidopsis, Eth inhibits taproot elongation through PIN2-mediated auxin-mediated IAA transport (Miao et al., 2018), BRs also regulate the plant gravity response by changing the polar transport of IAA (Li et al., 2005), JA controls lateral root formation by regulating auxin biosynthesis and polar transport (Sun et al., 2009), and phloem-mediated CK transport increases IAA biosynthesis (Jones et al., 2010) and polar transport (Bishopp et al., 2011). In rice, under low-phosphorus and low-nitrogen conditions, SL affects root development by altering the transport of IAA from stem to root (Sun et al., 2014).

The molecular mechanism determining the transport mode of phytohormones in grafted seedlings and their regulation after transport is poorly understood. As different combinations of rootstocks and scions have different modes of synthesis and transportation of endogenous hormones (Lacombe and Achard, 2016), further efforts are needed to reveal the metabolic levels and interaction mechanisms of various endogenous hormones in rootstocks and scions after vegetable grafting and to determine how to affect the expression of rootstock traits.

Phytohormone crosstalk is also involved in the regulation of growth in grafted vegetables; and GA and ABA have been shown to regulate growth in grafted tomato (Gaion et al., 2018). These studies suggest that phytohormones (ABA, IAA, and CK) may function as long-distance signals in grafted plants.

### Mineral Elements

Mineral elements are generally absorbed by the roots and then transported through the Casparian strip or by membrane transport proteins in the xylem (Thakur et al., 2016). Grafting has been the main method used to investigate ways to improve ion absorption, exclusion, and transport (Rouphael et al., 2008; Savvas et al., 2010b), as well as to confine heavy metal uptake and translocation in vegetables (Lux et al., 2011; Colla et al., 2013). Recently, several groups have reported that plants exposed to salt stress have altered gene expression patterns, metabolic activity, and ion and water transport, which reduces stress damage and restores water balance (Serrano and Gaxiola, 1994; Hasegawa et al., 2000; Si et al., 2010; Wang Q. et al., 2017). For example, it has been shown that *Cucurbita* rootstocks increase the plant’s tolerance to salt stress by excluding Na^+^ from leaf mesophyll cells and sequestering it in the leaf veins, retaining K^+^ in the leaf mesophyll cells, as well as by early ABA-induced stomatal closure (Niu et al., 2018b). The mechanism of ion (Na^+^, K^+^, V^+^, and Cu^2+^) exclusion has also been investigated in watermelon(*Citrullus lanatus*) rootstocks (Edelstein et al., 2011; Huang et al., 2013a, b; Nawaz et al., 2018) and cucumber(*Cucumis sativus*) rootstocks (Rouphael et al., 2008). In addition, grafting did not affect the uptake of Cd (cadmium) by the roots of grafted eggplant (*Solanum melongena*) (Arao et al., 2008) but did affect its transport in the xylem (Savvas et al., 2010a) and xylem loading (Mori et al., 2009).

In summary, the mechanisms by which grafting affects the absorption and transport of mineral elements in vegetables are controlled by the rootstock genotype or by rootstock–scion interactions, which can enhance the tolerance of grafted vegetables to high/low mineral element levels. Thus, grafting is one of the direct strategies used to overcome the effects of toxic heavy metals (Edelstein and Ben-Hur, 2018). However, studies of the corresponding genes or proteins are lacking (Shabala, 2003), and further studies are needed to identify the genes and proteins regulating the absorption, exclusion, and transport of toxic ions within grafted vegetables.

## PHLOEM-Mediated Systemic Transport of Macromolecular Signal Molecules in Rootstock-Grafted Vegetables

Signaling macromolecules such as RNAs, microRNAs, and proteins move between the rootstocks and scions of grafted horticultural crops during long-distance transport in the reconstructed vascular system. By participating in information exchange between rootstocks and scions, the processes of transcription, gene expression, and protein translation can not only regulate growth but also improve the adaptation of vegetables to the surrounding environment (Lucas et al., 2001; Harada, 2010; Goldschmidt, 2014; Notaguchi, 2015; Warschefsky et al., 2016; Ko and Helariutta, 2017). The translocation of DNA, mRNA, proteins, and siRNAs induced by grafting has become an active research topic (Haroldsen et al., 2012; Wu et al., 2013; Notaguchi and Okamoto, 2015).

### “Omics” Analyses of Macromolecule Signal Exchanges Induced by Grafting

In horticultural plants, different rootstocks can induce significant changes in physiological responses (Ren et al., 2018), photosynthesis (Xu et al., 2018), hormonal responses (Ren et al., 2018), environmental adaptation (Yu et al., 2017; Xu et al., 2018), and the sugars and aromatic flavors of the plants (Zhao et al., 2018). Extensive mRNA signaling networks have been reported in the model plants *A. thaliana* (Thieme et al., 2015) and *N. benthamiana* (Zhang W. et al., 2014) by bioinformatics and “omics” analyses. Using large-scale data analysis technologies (genome-wide transcriptomic and proteomic analyses), researchers can analyze differentially expressed genes (DEGs), differentially expressed miRNAs (DEMs), and differentially accumulated proteins (DAPs) in various grafted plants.

Genome-wide analysis has made it possible to explore the expression patterns of IAA-related transporter genes such as *ClLAX*, *ClPIN*, and *ClABCB* in grafted watermelon in response to abiotic stresses (e.g., salt, drought, and cold), providing insight into the possible roles of the encoded transporters (Yu et al., 2017). Transcriptome profiling has been widely applied to analyze the roles of DEGs/DEMs in graft compatibility and abiotic stress resistance using watermelon scions grafted to squash rootstocks (Xu et al., 2016; Ren et al., 2018). Proteomics can be used to analyze the major categories of proteins in different treatments to investigate the mechanisms of graft-enhanced stress tolerance in scions. For example, comparative proteomic analysis was used to show that cucumber scions grafted to *Momordica* rootstocks could respond to heat stress by differentially accumulating photosynthesis-related proteins (Xu et al., 2018).

Therefore, “omics” analyses are important techniques that can be used to unravel the differential accumulation and transport of macromolecular signals in grafted vegetables.

#### mRNAs

Combined with heterologous grafting methods, RNA sequencing in different species after grafting showed that >3,000 endogenous mRNAs migrate between the stock and scion in grafted cucumber and watermelon (Omid et al., 2007; Ham et al., 2009; Zhang W. et al., 2016). In tomato, studies showed that 347 mRNAs move into the stems of the parasite plant dodder (*Cuscuta* sp.) near the attachment region (LeBlanc et al., 2013; Kim et al., 2014). In addition, some mobile mRNAs have been shown to have biological functions. For example, *CmNACP* (Ruiz-Medrano et al., 1999), *CmGAIP* (Haywood et al., 2005), *StBEL5* (Banerjee et al., 2006), and *PFP-Let6* (Kim et al., 2001) affect plant growth and development (i.e., dwarfing, apical meristem development, tuber growth, and root development, respectively; (Table 1).

**TABLE 1:** Overview of mobile mRNAs and proteins that have been identified in grafted vegetables.

**Class**	**Name**	**Detection method**	**References**
mRNA	347 tomato mRNAs	cDNA libraries	Kim et al., 2014
	3546 *Cucurbita maxima* mRNAs	RNA-Seq SNPs	Zhang Z. et al., 2016
	*CmPP16*	RT-PCR	Xoconostle-Cázares et al., 1999
	*CmNACP*	RT-PCR	Ruiz-Medrano et al., 1999
	*SlPFP-LeT6*	*in situ* RT-PCR	Kim et al., 2001
	*CmGAIP*	RT-PCR	Haywood et al., 2005
	*StBEL5*	RT-PCR	Banerjee et al., 2006
	*SlPS*	RT-PCR	Zhang et al., 2018
Protein	CmPP1/PP2	Western Blot	Tiedemann and Carstens-Behrens, 1994
	CmPP16	Western Blot	Xoconostle-Cázares et al., 1999
	CmFT	Mass Spectrum	Lin et al., 2007
	CmRBP50	Gel mobility-shift assays	Ham et al., 2009
	SlCyp1	Western Blot	Spiegelman et al., 2015

*CmFT, Cucurbita moschata FLOWERING LOCUS T; CmPP16, Cucurbita maxima PHLOEM PROTEIN 1; CmNACP1, Cucurbita maxima NAC DOMAIN PHLOEM PROTEIN 1; SlPS, Solanum lycopersicum systemic protein precursor (prosystemin, PS); StBEL5, Solanum tuberosum BEL1-LIKE PROTEIN 5; CmGAIP, GIBBERELLIC ACID INSENSITIVE; SlPFP-LeT6, Solanum lycopersicum PYROPHOSPHATE-DEPENDENT PHOSPHOFRUCTOKINASE (PFP), and LeT6, a tomato KNOTTED-1–like homeobox (KNOX) gene, PFP-LeT6 fusion mRNA; CmRBP50, RNA Binding Protein 50; SlCyp1, Solanum lycopersicum Cyclophilin 1.*

Most mRNAs, but not their encoded proteins, are translated and function after transport. For example, tomato systemic protein precursor (prosystemin, *PS*) mRNA moves through the graft interface, after which it is unloaded to nucleated cells of the scion. *PS* mRNA’s ability to be translated into PS protein in response to external herbivore and pathogen damage – without the PS protein moving – demonstrates one function of mRNA transport (Zhang et al., 2018). Furthermore, long-distance transport of cyclophilin *SlCyp1* mRNA is associated with modulation of the root-shoot ratio, which responds to changes in light intensity by regulating root growth (Spiegelman et al., 2015). Finally, antiflorigen *PEBP* mRNA has been confirmed to be mobile and to inhibit flowering in tomato–tobacco heterografts (Huang et al., 2018).

#### ncRNAs

Many non-coding RNAs (ncRNAs), including transfer RNAs (tRNAs), siRNAs, and microRNAs (miRNAs), are found in the phloem sap of pumpkin(*Cucurbita moschata*) and oilseed rape (*Brassica napus* L.) (Buhtz et al., 2008; Pant et al., 2008; Zhang et al., 2009; Hu et al., 2016; Zhang W. et al., 2016), and their functions in virus defense, cell signal transmission, and gene expression regulation have been demonstrated.

#### tRNAs

Similar to other phloem-specific RNAs, tRNA molecules are selectively transferred into the sieve tubes (Yoo et al., 2004). Pumpkin phloem sap contains many full-length specific tRNA fragments that can interfere with ribosomal activity and effectively block translation. The tRNA fragments delivered to the phloem are potential long-distance signals; for example, cytokinin-containing tRNAs that can breakdown into free cytokinins are a source of CK (cytokinins) (Mok and Mok, 2001; Table 2). Non-coding RNAs (longer than si/miRNAs) that range from 30 to 90 nucleotides are also found in pumpkin phloem exudates and may inhibit protein translation (Zhang et al., 2009).

**TABLE 2:** Overview of mobile non-coding RNAs that have been identified in grafted vegetables.

**Crop**	**non-coding RNA**	**Transport direction**	**Function description**	**References**
*Solanum tuberosum* L.	miR172	shoot to root	Promote flowering, accelerates tuberization	Martin et al., 2009
*Brassica napus* L.	miR395	shoot to root	Sulfate starvation	Jones-Rhoades and Bartel, 2004
*Brassica napus* L.	miR398	shoot to root	Copper starvation	Sunkar et al., 2006
*Brassica napus* L.	miR399	shoot to root	Phosphate starvation	Pant et al., 2008
*Cucurbita maxima*	tRNA	shoot to root	The source of cytokinins	Mok and Mok, 2001

#### si/miRNAs

Zhang W. et al. (2014) reported that si/miRNAs function in systemic acquired resistance and mineral element uptake after they are transported through the phloem in both model plants and vegetables. Buhtz et al. (2010) showed that the mobility of miRNAs produced in rootstocks through the graft union helps the scion (miRNA processing *hen1-1* mutant, in this case) respond to nutrient deprivation. Nutrient starvation experiments showed that the levels of miR395, miR398, and miR399 in the phloem sap of *B. napus* were significantly increased in response to sulfate, copper, and phosphate starvation, respectively (Table 2). In particular, miR399 levels are markedly increased in low-phosphate medium, which is consistent with earlier studies in *A. thaliana* (Fujii et al., 2005; Bari et al., 2006; Chiou et al., 2006). CsmiR399a/e is upregulated to induce the tissue-specific transport of mRNAs under inorganic phosphate stress after grafting in cucumbers (Pant et al., 2008; Nawaz et al., 2016; Zhang Z. et al., 2016).

Visual markers can be used to track the distribution of siRNAs in the plant vascular system. Using asexual and sexual progeny of chimeras that consist of red cabbage (*B. oleracea* var. *capitata*) and tuber mustard (*Brassica juncea* var. *tumida*) after grafting (Li et al., 2013) showed that siRNAs are transported from the red cabbage to the tuber mustard, and that the transport of siRNAs created a heritable variation. Research on si/miRNA transportation provides a theoretical basis for using transgenesis to create an RNA interference vector to produce plants with reduced expression of related genes.

### Proteins

Numerous studies have shown that phloem sap contain proteins that can move not only between adjacent cells and distant cells, but also between the rootstock and scion in grafted plants. Functional mobile proteins that move between the rootstock and scion include the transport proteins CmPP1 and CmPP2 (Tiedemann and Carstens-Behrens, 1994; Golecki et al., 1999), the florigen CmFT (Lin et al., 2007), and CmSTMwhich determines cell fate in the meristem (Groot et al., 2005; Table 1).

At least nine additional phloem transport proteins, such as CmPP16 and CmHSP70, or their precursors, form a ribonucleoprotein (RNP) complex that is also present in the phloem of both the rootstock and the scion, indicating that the proteins are exchanged through the reconstructed vascular system (Golecki et al., 1998; Xoconostle-Cázares et al., 1999). A model in which RNP complexes based on CmRBP50 function in the translocation stream has been proposed for pumpkin phloem (Ham et al., 2009) and *B. napus* phloem sap (Ostendorp et al., 2017). Similar to CmRBP50, CmWRKYP, CmPP2, Cmlec17, and CmPP16 are phloem RNA-binding proteins that can also help RNA transport via phloem (Ham et al., 2009).

Taken together, analyses of phloem sap collected from cucumber, watermelon, pumpkin, and *B. napus* have revealed significant differences in the metabolism and proteome characteristics of shoot–root junction regions and those of the sieve tube system (STS) (Cho et al., 2015; Hu et al., 2016; Zhang W. et al., 2016; Ham and Lucas, 2017; Wang J. et al., 2017; Kehr and Kragler, 2018). These observations suggest that the STS is a distinct and extremely complex metabolic space in the plant vascular system that facilitates stock–scion communication.

### Studies on the Mechanism of RNA and Protein Signaling Between the Rootstock and Scion in Grafted Vegetables

**FIGURE 1:**
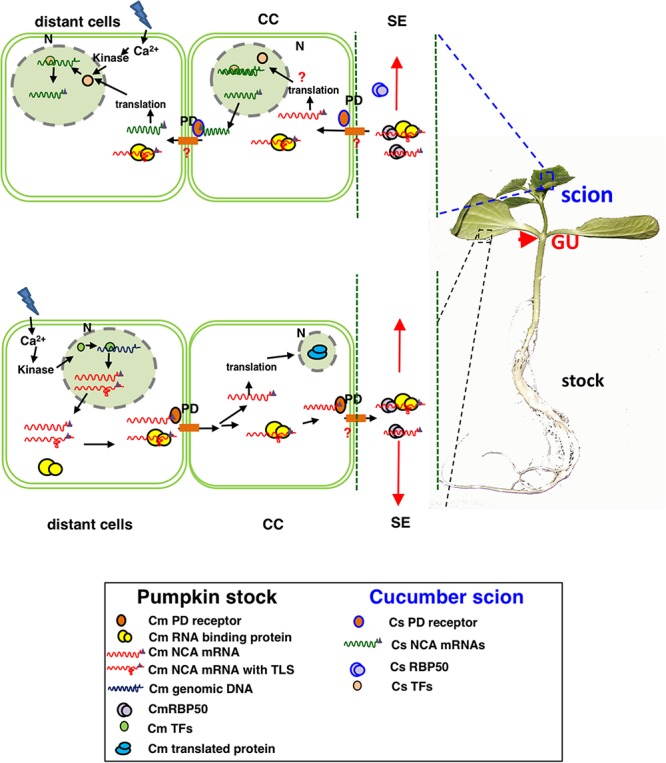
Proposed model for the selective movement of mRNA and protein from companion cells to sieve elements in grafted cucurbits. When grafted plants experience stress, the source tissues produce poly(A)-transcripts that are transcribed in companion cells, can be recognized by RNA-binding proteins, and selectively move through plasmodesmata into sieve elements. These mobile transcripts then bind with phloem proteins to form RNP complexes with specific motifs such as poly-CU, TLS, or 5-methylcytosine methylation sites. Furthermore, they are targeted to sink cells in the phloem flow and are unloaded into destination cells, where they are translated and function.

The mechanism of mRNA signaling in vegetable plants is not particularly well understood. It is uncertain whether the mechanism of mRNA transport is sequence-specific or non-sequence-specific. It has been reported that mRNA transport in *A. thaliana* may be (i) directional and tissue specific (Wang J. et al., 2017; Kehr and Kragler, 2018); (ii) independent of transcript abundance and stability (Calderwood et al., 2016); and (iii) facilitated by a specific tertiary structural sequence (Zhong et al., 2007), homodomain region (Kim et al., 2001), 3′-UTR (Haywood et al., 2005; Huang and Yu, 2009), poly-CU (Ham et al., 2009), TLS (tRNA-related sequence) (Zhang W. et al., 2016), and 5-methylcytosine modifications (Yang et al., 2019) that recognize cytoplasmic ribosomes and chaperones, altering RNA self-conformation and the plasmodesma exclusion limit (SEL), as well as by selective loading and unloading in the CC-SE (companion cell–sieve element) (Figure 1).

Transport of small RNA molecules via the phloem is mediated by the RNA-binding protein CmPSRP1 (Yoo et al., 2004). CmPSRP1 isolated from pumpkin phloem selectively binds to single-stranded siRNAs and mediates their intercellular trafficking through plasmodesmata, which is consistent with the function of CmPSRP1 in transporting siRNA through CC-SE plasmodesmata. These observations are related to the mechanism of siRNA-related gene silencing (Ham et al., 2009, 2014; Hu et al., 2016).

The mechanism of RNA and protein signaling between rootstock and scion in horticultural plants has been studied only in cucurbits, tomato, and *Brassica* species. Large-scale “omics” data analyses suggest that mRNA transport in grafted vegetables is directional and specific, and that the transcripts function after transport. Further studies that use gene-editing technology and focus on species-specific traits are needed.

## Discussion

Vegetables are exposed to various biotic and abiotic stresses that threaten production in open-field and greenhouse cultivation, in part because of continuous cropping (Cohen et al., 2000, 2005, 2007; Yu et al., 2017). Grafting has long been used to regulate plant growth and improve biotic and abiotic stress tolerance in the production of horticultural plants (Xu et al., 2018). Grafting has been studied for its striking effects on fruit quality (Bertucci et al., 2018; Zhao et al., 2018; Garcia-Lozano et al., 2020), physiological responses (Ren et al., 2018), and photosynthesis (Xu et al., 2018). Furthermore, heterografting is a powerful tool for identifying horizontal DNA movement (Yu et al., 2017) and the long-distance transport of mRNAs (Banerjee et al., 2006; Ham et al., 2009; Xu et al., 2016; Huang et al., 2018), ncRNAs (Mok and Mok, 2001; Yoo et al., 2004; Zhang et al., 2009; Ren et al., 2018), and proteins (Lin et al., 2007; Xu et al., 2018). Compared with those done in fruit trees, there are fewer studies on the physiological and molecular aspects of rootstock–scion interaction in vegetables. Currently, grafting is principally used in the *Cucurbitaceae* (watermelon, melon, cucumber) and *Solanaceae* (tomato, pepper, eggplant) families and has become a more commonly used and more efficient technique for studies aimed at elucidating the underlying mechanism(s) of the rootstock–scion interaction in vegetables than grafting for such studies in fruit trees. First, vegetables are herbaceous plants with short growth cycles (<2 years per growth cycle), so it is more convenient to investigate phenotypic changes during the growth and development of vegetables than during those of fruit trees. Second, owing to the high heterozygosity of germplasm resources in fruit trees, it is easier to obtain homozygous vegetable plants whose gene sequence is stable, which are advantageous for studies on rootstock–scion signaling exchanges. Third, gene modification/editing technology and ethyl methanesulfonate (EMS) mutation screen technology have been established in vegetables, especially in *Cucurbitaceae* (watermelon, cucumber) and *Solanaceae* (tomato), which offers more opportunity to study rootstock–scion signaling exchange and interaction by grafting mutants to transgenic plants. In a word, there is an urgent need for studies on rootstock breeding and the rootstock–scion interaction in grafted vegetables.

**FIGURE 2:**
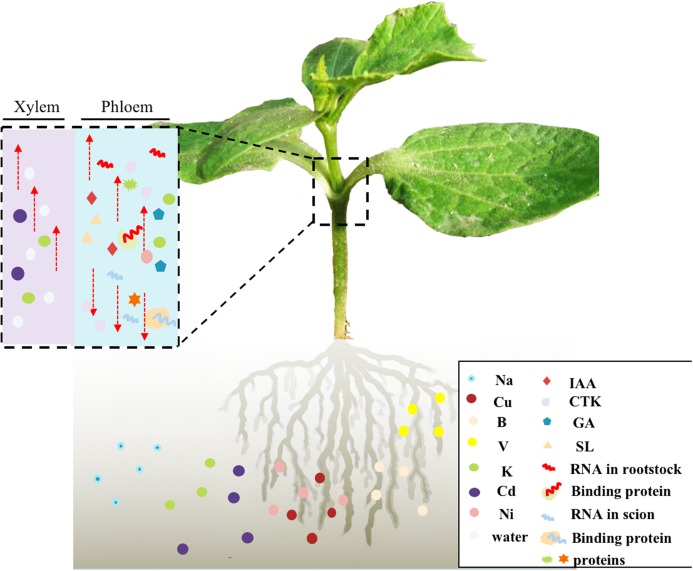
Proposed model of systemic long-distance signal transport in distant tissues of grafted vegetables. Grafting material: cucumber/pumpkin cotyledon-insertion grafts. Directionality: mineral elements and water acquired by the rootstock can be transported to the scion through the xylem; and plant hormones, some microRNAs, and proteins can be transported through the phloem rootstock–scion communication pathway in grafted vegetables. In addition, some binding proteins can transport RNAs. Selectivity: rootstocks of grafted vegetables will selectively absorb or reject mineral elements in the rhizosphere so as to achieve tolerance of grafted vegetables to high/low mineral element levels.

Unraveling the mechanisms that control vascular-delivered signaling molecules in grafted vegetables would enable the selection of suitable rootstock resources and also help to explain how grafting improves quality in vegetables. Several studies have focused on the changes in and transport of phytohormones (Noorden et al., 2006; Hirose et al., 2008), minerals (Huang et al., 2013b; Niu et al., 2018a), mRNAs (Omid et al., 2007; Ham et al., 2009; Zhang W. et al., 2016), ncRNAs (Buhtz et al., 2008; Pant et al., 2008), and proteins (Kehr and Kragler, 2018; Xu et al., 2018) that are induced by grafting. Some phytohormones (Ko et al., 2014; Ivanchenko et al., 2015; Regnault et al., 2015; Guo et al., 2016), mRNAs (Haywood et al., 2005; Banerjee et al., 2006), ncRNAs (Li et al., 2013), and proteins (Groot et al., 2005; Ham et al., 2009) that can be transported via the vascular system have been proven to have definite functions (Figure 2). Researchers have speculated about the mechanisms of RNA and protein phloem transport/co-transport, in regard to tissue specificity (Wang J. et al., 2017; Kehr and Kragler, 2018), whether specific structural sequences are required (Kim et al., 2001; Haywood et al., 2005; Huang and Yu, 2009; Ham et al., 2009; Zhang W. et al., 2016; Yang et al., 2019), and the role of RNA-binding proteins (Yoo et al., 2004; Ham et al., 2009; Ostendorp et al., 2017). Generally, research on grafted vegetables has mainly focused on stress resistance (Rouphael et al., 2008; Yu et al., 2017), graft compatibility (Ren et al., 2018), hormonal responses (Albacete et al., 2009; Gaion et al., 2018), and long-distance transport signals in vascular systems (Lin et al., 2007; Li et al., 2013; Zhang et al., 2018; Huang et al., 2018). Nevertheless, “omics” analyses are direct and rapid methods of detecting and identifying mobile and differentially expressed substances (Yu et al., 2017), such as DEGs (Xu et al., 2016), DEMs (Ren et al., 2018), and DAPs (Xu et al., 2018) in grafted vegetables; however, results obtained from “omics” datasets need to be verified by combining more biological experiments, such as fluorescence quantitative analysis of gene expression pattern.

## Conclusion

Despite the current state of research, the mechanisms that control the changes in and transport of specific molecules in vegetable grafts remain poorly understood. In particular, the basic questions (what, when, where, and how) concerning the transport of signal substances between rootstock and scion remain to be addressed and merit further study. Moreover, a scientific explanation for the long-distance trafficking of RNAs and proteins in rootstock grafts remains to be determined. This explanation will also provide a theoretical basis for rootstock breeding and selection to meet specific demands for efficient vegetable production.

## Author Contributions

XHL and WZ wrote the final manuscript. WL wrote mRNA and protein part. TW wrote non-coding RNA part. JZ wrote nutrient transport part. XJL wrote hormone transport part.

## Conflict of Interest

The authors declare that the research was conducted in the absence of any commercial or financial relationships that could be construed as a potential conflict of interest.

## Abbreviations

IAA: indole acetic acid (an auxin)
CL: carlactone
ABA: abscisic acid
GA: gibberellic acid
CK: cytokinins
ACC: 1-aminocyclopropanecarboxylic acid
JA: jasmonic acid
SL: strigolactone
*t*Z: *trans*-zeatin
*t*ZR: *trans*-zeatin riboside
DEGs: differentially expressed genes
DEMs: differentially expressed miRNAs
DAPs: differentially accumulated proteins.
